# Health-Related Quality of Life in Pediatric Patients with Syndromic Autism and their Caregivers

**DOI:** 10.1007/s10803-021-05030-8

**Published:** 2021-05-03

**Authors:** Corneliu Bolbocean, Fabiola N. Andújar, Maria McCormack, Bernhard Suter, J. Lloyd Holder

**Affiliations:** 1 Department of Preventive Medicine, University of Tennessee Health Science Center, Memphis, TN USA; 2 The Centre for Addiction and Mental Health, Toronto, ON Canada; 3 Jan and Dan Duncan Neurological Research Institute, Texas Children’s Hospital, 1250 Moursund St. Suite 925, Houston, TX 77030 USA; 4 Division of Neurology and Developmental Neuroscience, Department of Pediatrics, Baylor College of Medicine, Houston, TX USA; 5 Nuffield Department of Primary Care Health Sciences, Oxford University, Radcliffe Primary Care Building, Radcliffe Observatory Quarter, Woodstock Rd, Oxford, OX2 6GG USA

**Keywords:** Autism spectrum disorder, Clinical research, Diabetes, Idiopathic autism, Intellectual disability, Pediatric quality of life inventory, Phelan-McDermid syndrome, Rett syndrome, *SYNGAP1* related intellectual disability, Health related quality of life, Beach center family quality of life

## Abstract

Children with autism have a significantly lower quality of life compared with their neurotypical peers. While multiple studies have quantified the impact of autism on health-related quality of life (HRQoL) through standardized surveys such as the PedsQL, none have specifically investigated the impact of syndromic autism. Here we evaluate HRQoL in children diagnosed with three genetic disorders that strongly predispose to syndromic autism: Phelan-McDermid syndrome (PMD), Rett syndrome (RTT), and *SYNGAP1*-related intellectual disability (*SYNGAP1*-ID). We find the most severely impacted dimension is physical functioning. Strikingly, syndromic autism results in worse quality of life than other chronic disorders including idiopathic autism. This study demonstrates the utility of caregiver surveys in prioritizing phenotypes, which may be targeted as clinical endpoints for genetically defined ASDs.

## Introduction

The diagnosis of autism spectrum disorders (ASDs) has increased dramatically over the past decade (Zablotsky et al., 2019). The current USA prevalence is 1 in 59 children (1.7%) by the age of 8, with boys diagnosed 4 to 5 times more often than girls (Maenner et al., 2020). Diagnostic criteria for ASDs have been revised in the most recent diagnostic and statistical manual of mental disorders, 5th edition (DSM-5) (Lee et al., 2015). Autism is diagnosed if the two main criteria are met: persistent deficits in social communication and restrictive or repetitive patterns of behaviour. Importantly, two qualifiers must also be present: the onset of symptoms must be early in development and the symptoms, particularly delayed communication, must be greater than expected for impairments in other developmental domains.

Autism spectrum disorders are defined as either syndromic or non-syndromic. Children diagnosed with non-syndromic ASD meet the DSM-5 criteria without other significant somatic or neurologic manifestations. This constitutes the majority of individuals diagnosed with autism. In contrast, syndromic ASDs are diagnosed in individuals meeting DSM-5 criteria but also manifesting somatic symptoms or additional neurologic phenotypes. The somatic symptoms may be facial or other physical dysmorphic features or more complex congenital organ abnormalities. Neurologic diagnoses commonly co-occurring with ASD include intellectual disability and epilepsy (Besag & Vasey, 2020; Thurm et al., 2019). Children with a clinical diagnosis of syndromic autism are more likely to have a genomic abnormality than individuals with non-syndromic ASD (Ashitha & Ramachandra, 2020; Tammimies et al., 2015).

Prolonged and multidimensional clinical issues associated with syndromic ASDs are linked to negative lifelong health and socio-economic impact of the affected child and caregivers (Marsack-Topolewski & Church, 2019; Ten Hoopen et al., 2020). Thus, understanding the health-related quality of life (HRQoL) in children with syndromic ASD and their caregivers is critical to design clinically effective and economically viable interventions for these children.

Health-related quality of life is a multidimensional concept designed to directly measure an individual’s states related to physical, psychological, and social cognitive aspects of life (Cummins, 2005). Literature on HRQoL in children has identified a lower HRQoL in autism relative to other chronic disorders. However, very few studies have examined the HRQoL in pediatric patients diagnosed with syndromic autism. Two studies reported that children with fragile X syndrome have significantly impaired health-related quality of life with cognitive function most affected (Coffman et al., 2020; Fitzpatrick et al., 2020). HRQoL for other genetic disorders predisposing to syndromic autism such as Rett syndrome (RTT), Phelan-McDermid syndrome (PMD) and *SYNGAP1*-related intellectual disability (*SYNGAP1*-ID)*,* are not known yet.

The main objective of this study was to evaluate for the first time the HRQoL of children diagnosed with RTT, PMD and *SYNGAP1*-ID*,* using the PedsQL 4.0 Inventory (see Appendix A for more details of clinical phenotypes associated with each disorder). Our study’s secondary objective was to measure the differential impact on HRQoL in children with RTT, PMD and *SYNGAP1*-ID and their caregivers. We wanted to determine the minimum number of variables which could describe the observed variation in PedsQL and FQOL measures. This analysis might provide useful insights into the design of targeted interventions aimed at improving HRQoL in patients and caregivers.

This study combines data from multiple genetic disorders that frequently result in syndromic autism for the first time, to broadly approximate the impact of syndromic autism on HRQoL in children and caregivers. Moreover, we are able to compare HRQoL between each of these genetically-defined disorders and other chronic childhood illnesses to discover their impact on affected children and their families.

## Methods

### Participants

This study employed a cross-sectional design. Participants included 391 families with children diagnosed with genetic disorders which strongly predispose to autism; 213 (54.5%) of the participants were diagnosed with PMD, 148 (37.9%) with RTT, and 30 (7.7%) with *SYNGAP1*-ID. Inclusion criteria consisted of children 2–18 years of age with an ASD diagnosis of PMD, RTT or *SYNGAP1*-ID. For recruitment, an email providing access to an online qualtrics survey was provided to families with self-identified interest in participation through the following organizations: Phelan-McDermid Syndrome Foundation, RettSyndrome.org, or bridge the gap: *SYNGAP1* Education and Research Foundation. Patients were recruited from throughout the USA. This study did not collect socio-demographic information related PMD, RTT and *SYNGAP1* patients. However, study participants were selected using Phelan-McDermid Syndrome Foundation, RettSyndrome.org, and bridge the gap: *SYNGAP1* patient databases. Appendix B provides socio-demographic information related to PMD, and *SYNGAP1* patients based on above referenced patient databases (data was not available for RTT).

### Procedure

Approval for the study was obtained from Baylor College of Medicine’s Institutional Review Board. All data was ascertained through caregiver self-report questionnaires. For the purposes of our study, the pediatric quality of life inventory^™^ version 4.0 (PedsQL) was administered as a quantifiable tool with which to assess the health-related quality of life of participant children. The Beach center family quality of life scale (FQOL) questionnaire was also administered and assessed parents’ life satisfaction with their nuclear family. The surveys were estimated to take approximately 15 min to complete. They were anonymous, and the only other information caregivers were requested to provide was their child’s diagnosis. No compensation was provided.

### PedsQL

The PedsQL is one of the most widely studied assessments of child HRQoL reports (Bastiaansen et al., 2004; Cummins, 2005; Fitzpatrick et al., 2020; Killedar et al., 2020; Stokes et al., 2017). As an assessment instrument, it provides a modular approach in measuring health-related quality of life (HRQoL) in healthy children as well as those with acute or chronic conditions across four domains: physical, emotional, social, and school functioning. These domains have been identified as core dimensions of health by the World Health Organization (Bastiaansen et al., 2004). These dimensions are reflected in the survey as four scales comprising physical (8 items), emotional (5 items), social (5 items), and school functioning (5 items). From these four core scales, a total quality of life score (based on all items) was calculated (Killedar et al., 2020). The survey consisted of 23 items in the format: “In the past ONE month, how much of a problem has your child had with…,” rated on a five-point Likert scale: (0 = Never a problem, 1 = Almost never a problem, 2 = Sometimes a problem, 3 = Often a problem, 4 = Almost always a problem).

All items in the PedsQL were reverse scored and linearly transformed to a 0 to 100 scale, with higher scores indicative of greater HRQoL per standard reporting practice. Consistent with standard scoring practices, scores were transformed to (0 = 100, 1 = 75, 2 = 50, 3 = 25, and 4 = 0), and means were computed for each domain. To calculate the total scale score for each disorder, we computed the mean as the sum of all items across all modules. We focused our subsequent analysis on the PMD and RTT data sets due to the relatively small number of *SYNGAP1*-ID participants.

### FQOL

The beach center family quality of life (FQOL) Scale is a standardized instrument that is a validated measure of quality of life for families with children (Liu et al., 2020; Perry & Isaacs, 2015; Schlebusch et al., 2017). Several studies have demonstrated the suitability of the beach center FQOL scale to assess the quality of life of families of children with disabilities. Moreover, this instrument had been successfully used to assess family quality of life with preschool-aged children diagnosed with ASD (Rivard et al., 2017; Schlebusch et al., 2017).

The Beach Center FQOL Scale was administered as a 25-item inventory utilizing satisfaction as the primary response format with 5 dimensions: family interaction, parenting, emotional well-being, physical/material well-being, and disability-related support. Response options were rated on a five-point Likert scale: (1 = very dissatisfied, 2 = dissatisfied, 3 = neither satisfied nor dissatisfied, 4 = satisfied, and 5 = very satisfied).

### Analysis

Participants were divided into three groups based on genetic diagnosis. We implemented parametric and non-parametric methods to test for mean differences across PedsQL and FQOL dimensions between groups (Tables 1 and 2). We removed *SYNGAP1*-ID participants from further analysis due to relative small sample size and tested for differences in HRQoL in patients and caregivers between RTT and PMD. We performed a permutation test as robustness check.

**Table 1:** PedsQL summary

DOMAINS	Combined syndromic ASDs N = 391 (St. Dev.)	PMD N = 213 (St. Dev.)	RTT N = 148 (St. Dev.)	*SYNGAP1*-ID N = 30 (St. Dev.)	Kruskal–Wallis H test p-value
PHYSICAL	33.55 (11.09)	42.09 (15.51)	19.64 (9.35)	38.92 (9.94)	0.001
1. Problems with walking more than one block	45.44 (19.45)	61.03 (15.53)	23.64 (4.43)	51.67 (3.16)	0.001
2. Problems with running	33.44 (16.59)	47.64 (8.40)	15.20 (3.39)	37.5 (4.85)	0.001
3. Problems with participating in sports activities or exercise	27.21 (10.20)	34.43 (3.55)	15.54 (2.93)	31.67 (5.05)	0.001
4. Problems with lifting something heavy	29.76 (14.38)	37.32 (4.15)	13.17 (4.16)	38.79 (2.28)	0.001
5. Problems with taking a bath or shower by him or herself	19.06 (6.42)	19.95 (4.86)	12.24 (4.80)	25.00 (8.16)	0.046
6. Problems with doing chores around the house	21.53 (9.97)	24.06 (1.79)	10.54 (4.19)	30.00 (5.09)	0.001
7. Problems with having hurts or aches	46.88 (11.35)	54.97 (7.05)	33.90 (6.05)	51.79 (4.62)	0.001
8. Problems with low energy level	45.09 (12.20)	57.34 (7.05)	32.93 (6.39)	45.00 (4.24)	0.001
EMOTIONAL	55.13 (10.24)	62.14 (10.96)	51.59 (7.67)	51.65 (14.14)	0.001
1. Problems with feeling afraid or scared	56.63 (9.23)	67.18 (11.19)	52.73 (8.54)	50.00 (4.36)	0.001
2. Problems with feeling sad or blue	56.66 (6.84)	64.33 (9.75)	51.19 (9.80)	54.46 (6.66)	0.001
3. Problems with feeling angry	51.83 (9.74)	59.38 (8.29)	55.27 (10.38)	40.83 (7.62)	0.001
4. Problems with trouble sleeping	41.03 (3.60)	45.18 (5.14)	39.11 (5.52)	38.79 (2.59)	0.169
5. Problems with worrying about what will happen to him or her	69.48 (8.52)	74.63 (22.09)	59.64 (9.68)	74.17 (5.39)	0.001
SOCIAL	39.83 (24.47)	38.45 (21.51)	37.71 (29.57)	43.34 (23.06)	0.001
1. Problems with getting along with other children	53.93 (6.10)	50.00 (5.92)	60.95 (11.16)	50.83 (2.12)	0.004
2. Problems with other kids not wanting to be his or her friend	45.37 (5.50)	41.99 (4.31)	42.41 (4.42)	51.72 (2.05)	0.333
3. Problems with getting teased by other children	69.56 (3.49)	65.70 (11.61)	70.48 (13.21)	72.5 (5.15)	0.414
4. Problems with not being able to do things other children his or her age can do	9.42 (4.48)	11.49 (1.03)	4.28 (0.40)	12.5 (8.67)	0.001
5. Problems with keeping up when playing with other children	20.90 (9.55)	23.09 (1.76)	10.44 (1.96)	29.16 (5.15)	0.001
SCHOOL	40.39 (15.05)	43.60 (16.34)	38.57 (10.61)	39.00 (18.78)	0.063
1. Problems with paying attention in class	27.58 (3.12)	28.29 (3.16)	30.29 (4.98)	24.17 (5.10)	0.001
2. Problems with forgetting things	32.92 (5.12)	33.58 (1.96)	37.69 (5.74)	27.5 (5.34)	0.363
3. Problems with keeping up with schoolwork	28.46 (4.75)	33.89 (4.56)	26.51 (3.53)	25.00 (5.34)	0.195
4. Problems with missing school because of not feeling well	60.09 (7.47)	64.65 (11.76)	51.47 (6.30)	64.17 (3.39)	0.001
5. Problems with missing school to go to the doctor or hospital	52.89 (5.45)	57.59 (7.15)	46.92 (6.33)	54.17 (4.30)	0.002

**Table 2:** FQOL summary

DOMAINS	Combined syndromic ASDs N = 391 (St. Dev.)	PMD N = 213 (St. Dev.)	RTT N = 148 (St. Dev.)	*SYNGAP1*-ID N = 30 (St. Dev.)	Kruskal–Wallis H test p-value
FAMILY INTERACTION	3.296 (0.940)	3.33 (0.886)	3.36 (0.970)	2.69 (0.932)	0.003
1. My family enjoys spending time together	3.402 (1.303)	3.385 (1.252)	3.520 (1.353)	2.933 (1.337)	0.124
2. My family members talk openly with each other	3.240 (1.264)	3.371 (1.220)	3.184 (1.282)	2.600 (1.303)	0.009
3. Our family solves problems together	3.195 (1.233)	3.288 (1.171)	3.223 (1.282)	2.400 (1.163)	0.003
4. My family members support each other to accomplish goals	3.189 (1.211)	3.178 (1.200)	3.311 (1.200)	2.667 (1.241)	0.038
5. My family members show that they love and care for each other	3.742 (1.199)	3.723 (1.163)	3.905 (1.163)	3.067 (1.413)	0.153
6. My family is able to handle life’s ups and downs	3.003 (1.201)	3.033 (1.203)	3.061 (1.191)	2.500 (1.167)	0.075
PARENTING	3.04 (0.846)	3.06 (0.847)	3.11 (0.836)	2.57 (0.769)	0.004
7. My family members help the children be independent	2.827 (1.227)	2.920 (1.204)	2.841 (1.217)	2.100 (1.242)	0.064
8. My family members help the children with schoolwork and activities	2.807 (1.216)	2.887 (1.222)	2.767 (1.145)	2.433 (1.455)	0.167
9. My family members teach the children how to get along with each other	3.189 (1.201)	3.175 (1.208)	3.315 (1.190)	2.667 (1.093)	0.087
10. Adults in our family teach the children to make good decisions	3.348 (1.172)	3.343 (1.156)	3.428 (1.159)	3.000 (1.313)	0.554
11. Adults in my family know other people in my children’s lives (friends, teachers, etc.)	3.209 (1.292)	3.192 (1.283)	3.310 (1.288)	2.833 (1.341)	0.084
12. Adults in my family have time to take care of the individual needs of every child	2.881 (1.291)	2.850 (1.280)	3.021 (1.310)	2.433 (1.194)	0.015
EMOTIONAL WELL-BEING	2.64 (0.904)	2.67 (0.931)	2.67 (0.895)	2.266 (0.659)	0.063
13. My family has the support we need to relieve stress	2.584 (1.289)	2.547 (1.274)	2.748 (1.302)	2.033 (1.189)	0.120
14. My family members have friends or others who provide support	2.700 (1.215)	2.708 (1.250)	2.689 (1.154)	2.700 (1.291)	0.352
15. My family members have some time to pursue our own interests	2.522 (1.318)	2.613 (1.346)	2.524 (1.281)	1.867 (1.137)	0.075
16. My family has outside help available to us to take care of special needs of all family members	2.767 (1.351)	2.831 (1.314)	2.735 (1.386)	2.467 (1.432)	0.062
PHYSICAL/MATERIAL WELL-BEING	3.67 (0.930)	3.741 (0.903)	3.59 (0.963)	3.43 (0.9305)	0.177
17. My family gets medical care when needed	3.828 (1.211)	3.882 (1.191)	3.764 (1.258)	3.767 (1.135)	0.234
18. My family has dental care when needed	3.723 (1.299)	3.849 (1.226)	3.547 (1.387)	3.700 (1.291)	0.164
19. My family members have transportation to get to the places they need to be	3.549 (1.325)	3.646 (1.289)	3.439 (1.386)	3.400 (1.248)	0.334
20. My family has a way to take care of our expenses	3.296 (1.303)	3.363 (1.322)	3.260 (1.254)	3.000 (1.390)	0.231
21. My family feels safe at home, work, school, and in our neighborhood	3.931 (1.164)	3.948 (1.174)	3.973 (1.140)	3.600 (1.192)	0.370
DISABILITY RELATED SUPPORT	3.11 (0.991)	3.1470 (0.316)	3.13 (1.031)	2.79 (1.018)	0.202
22. My family member with a disability has support to accomplish goals at school or at workplace	3.244 (1.259)	3.394 (1.290)	3.112 (1.175)	2.793 (1.292)	0.015
23. My family member with a disability has support to accomplish goals at home	3.216 (1.270)	3.268 (1.266)	3.221 (1.250)	2.833 (1.367)	0.257
24. My family member with a disability has support to make friends	2.784 (1.276)	2.739 (1.251)	2.885 (1.312)	2.600 (1.276)	0.502
25. My family has good relationships with the service providers who provide services and support to our family members with a disability	3.324 (1.307)	3.303 (1.328)	3.419 (1.278)	3.000 (1.287)	0.207

We were interested to determine the minimum number of variables or factors which could describe the observed variation in PedsQL and FQOL measures. Thus, we conducted the principal components analyses for the PedsQL and FQOL, separately for Rett and Phelan-McDermid syndrome patients and their caregivers in order to identify the minimum number of uncorrelated variables the observed variance of the dataset can be described. All analyses were conducted in STATA 15.

## Results

**Fig. 1:**
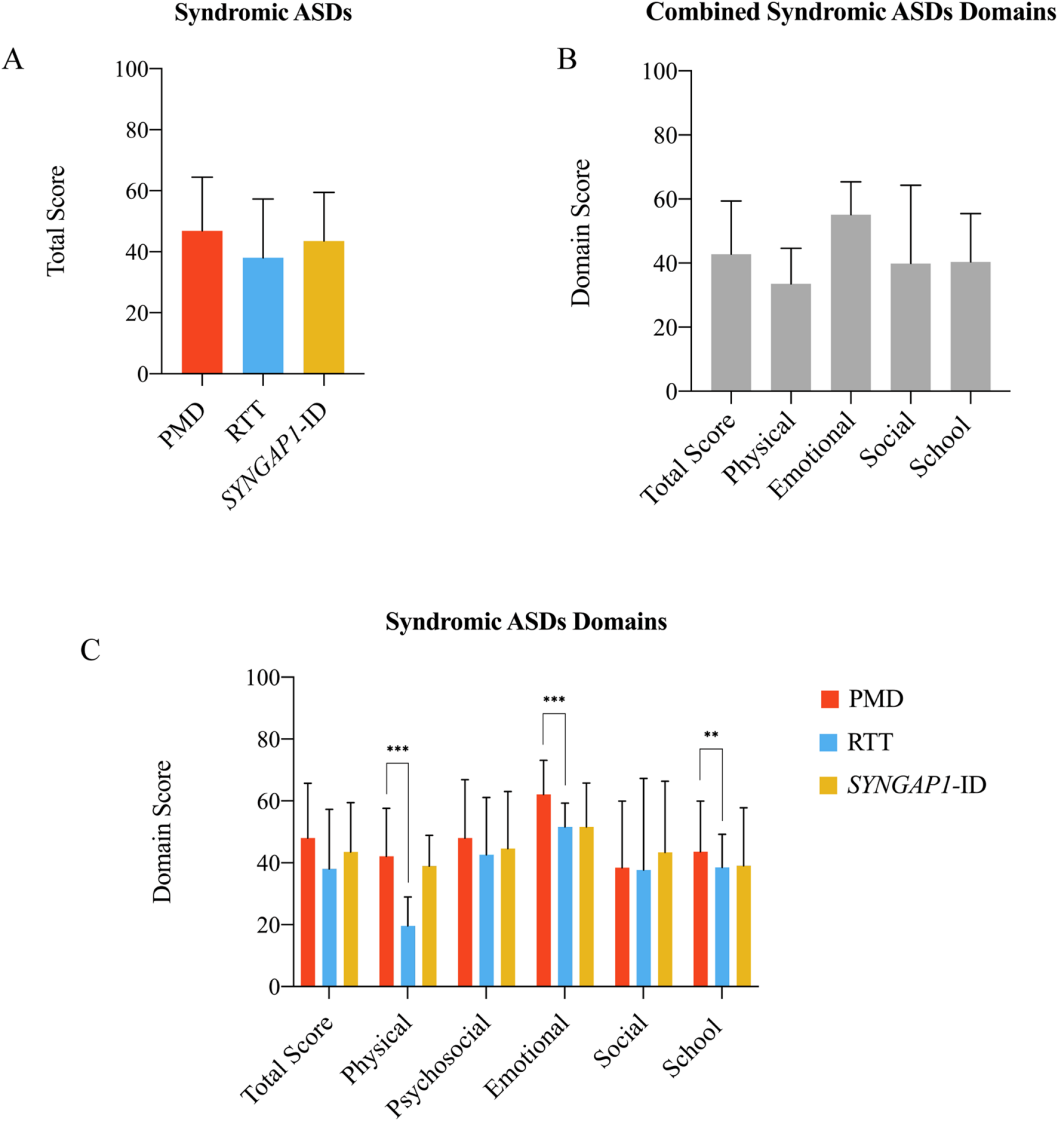
PedsQL data from Phelan-McDermid syndrome, Rett syndrome, and *SYNGAP1*-related intellectual disability. **a** Combined total scores for PMD, RTT and *SYNGAP1*-ID. **b** Total score and individual domain scores for combined syndromic autism spectrum disorders. C) Total score and individual domain scores for PMD, RTT and *SYNGAP1*-ID. ** p < 0.01, *** p < 0.001 (student’s t-test)

The PedsQL survey results revealed significant differences in health-related quality of life among the three syndromic autisms in each of the dimensions tested and in the overall score (Table 1 and Fig. 1a). RTT girls had the lowest total HRQoL score among our genetic disorders (M = 38.03), followed by *SYNGAP1-*ID (M = 43.51), and PMD (M = 46.87). Across dimensions, the greatest impairment in syndromic ASDs was in physical functioning (M = 33.55) (Fig. 1b). When parsing the data based on genetic diagnosis, the greatest impairment for PMD was social functioning (M = 38.45), while the greatest impairment for RTT was physical functioning (M = 19.64) (Fig. 1c and Table 1).

**Fig. 2:**
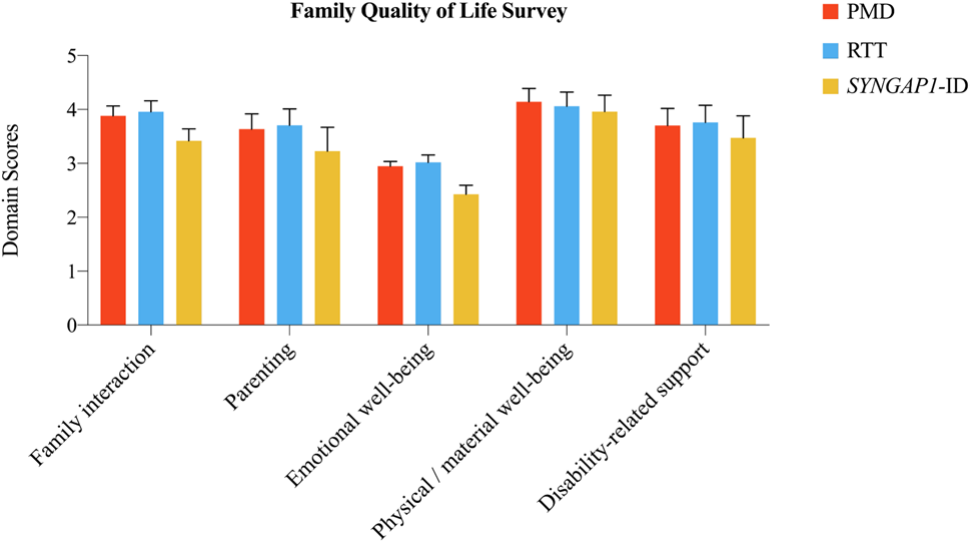
Beach center family quality of life scale domain scores

For FQOL (Table 2 and Fig. 2), the average scores of FQOL for combined syndromic autism for the family interaction (M = 3.75), parenting (M = 3.52) and disability-related support (M = 3.64) were between satisfied and neither satisfied or dissatisfied. For emotional well-being, the average score for combined syndromic autism was between dissatisfied and neither satisfied or dissatisfied (M = 2.79). Finally, for physical/material well-being, the average score for combined syndromic autism was in the satisfied range (M = 4.05). Thus, the greatest toll on family quality of life for syndromic autism is on emotional well-being. There were significant differences among the genetic disorders for parenting and family interaction which were driven by lower scores in the *SYNGAP1*-ID families. Direct comparison between RTT and PMD families revealed no significant differences for genetically-defined syndromic autisms demonstrating each has a similar impact on family quality of life.

We next directly evaluated for differences between PMD and RTT patients in the PedsQL dimensions (Fig. 1c). Overall, RTT patients scored worse than PMD across physical and emotional dimensions (p < 0.001). To further investigate the robustness of this finding, we performed a permutation test. In Table 3, we report p values from a two-sided permutation test of zero effect. In particular, we randomly assigned the diagnosis status (RTT, PMD) 10,000 times and computed the differences in average PedsQL scores across domains between two groups of patients (these estimates are called placebo estimates). Under random assignment of diagnosis there should be no difference in average PedsQL scores between RTT and PMD, i.e. the effect of diagnosis is by design not statistically significantly different from zero. The permutation test counts how many times the placebo estimates of the difference between scores are as extreme or more extreme than the observed differences. Thus, under the null hypothesis of zero difference between groups, the proportion of resampled differences that are greater or equal in absolute value than the observed difference provides a p-value for the null hypothesis. The results of the permutation test demonstrates that our main finding is robust. Thus, the overall evidence supports the conclusion that RTT patients compared to PMD patients score significantly worse across all PedsQL domains.

**Table 3:** Robust inference for differences between PMD and RTT PedsQL average scores

PedsQL domain	PMD N = 213	RTT N = 148	Difference	*t* test (d.f.)	p-value	Permutation test p-value
Average physical	4.19	3.23	0.964***	9.372 (359)	0.001	0.001
Average emotional	2.93	2.51	0.419***	5.190 (358)	0.001	0.001
Average social	3.48	3.47	0.007	0.090 (358)	0.982	0.889
Average school	3.46	3.25	−0207**	−2.021 (358)	0.043	0.062

*indicates a p-value less than 0.05

**p < 0.01; ***p < 0.001

*d.f.* stands for degrees of freedom

We next assessed the relationship between each of the PedQL dimensions using correlation analysis and tested for inter-item reliabilities for each factor using Cronbach’s alpha. We found a good level of reliability (Tables 4, 5, 6) with physical and emotional functioning scores having the highest reliability. Furthermore, for PMD patients, the highest correlations were between physical and school domains as well as social and school domains. In contrast for RTT patients, the correlations across domains were generally lower compared to PMD patients. The highest correlations for RTT were between the physical and school domains as well as between emotional and school domains. The high correlation between physical and school domains likely reflects difficulty in school participation due to physical impairment in girls with RTT.

**Table 4:** PedsQL correlations and inter-item reliabilities across all study measures and subscales PMD patients

Dimension	1	2	3	Cronbach’s alpha reliability
1. Physical	1			0.798
2. Emotional	0.30***	1		0.792
3. Social	0.33***	0.42***	1	0.748
4. School	0.47***	0.39***	0.50***	0.715

***p < 0.001

**Table 5:** PedsQL correlations and inter-item reliabilities across all study measures and subscales RTT patients

Dimension	1	2	3	Cronbach’s alpha reliability
1. Physical	1			0.65
2. Emotional	0.110***	1		0.67
3. Social	0.273***	0.32***	1	0.68
4. School	0.37***	0.25***	0.29***	0.67

***p < 0.001

**Table 6:** PedsQL correlations and inter-item reliabilities across all study measures and subscales PMD and RTT patients

Dimension	1	2	3	Cronbach’s alpha reliability
1. Physical	1			0.75
2. Emotional	0.31***	1		0.75
3. Social	0.29***	0.38***	1	0.73
4. School	0.43***	0.37***	0.42***	0.68

***p < 0.001

We next conducted principal components analyses for children’s PedsQL and caregivers’ FQOL (Table 7) separately by diagnosis and with all diagnoses combined. These analyses indicated that the number of factors for PedsQL and FQOL data matrices could be reduced into four to six factors and the percentage of explained variance was between 55 and 73%. This suggests that these individual dimension scores contain information, which might be truly heterogeneous in its nature for RTT and PMD patients and requires further investigation.

**Table 7:** Principal component analysis

	Number of factors with eigenvalue > 1	Cumulative variance explained (%)
PedsQL PMD	6	67
PedsQL RTT	6	73
PedsQL PMD and RTT	5	64
FQOL PMD	6	62
FQOL RTT	4	55
FQOL PMD and RTT	5	56

**Fig. 3:**
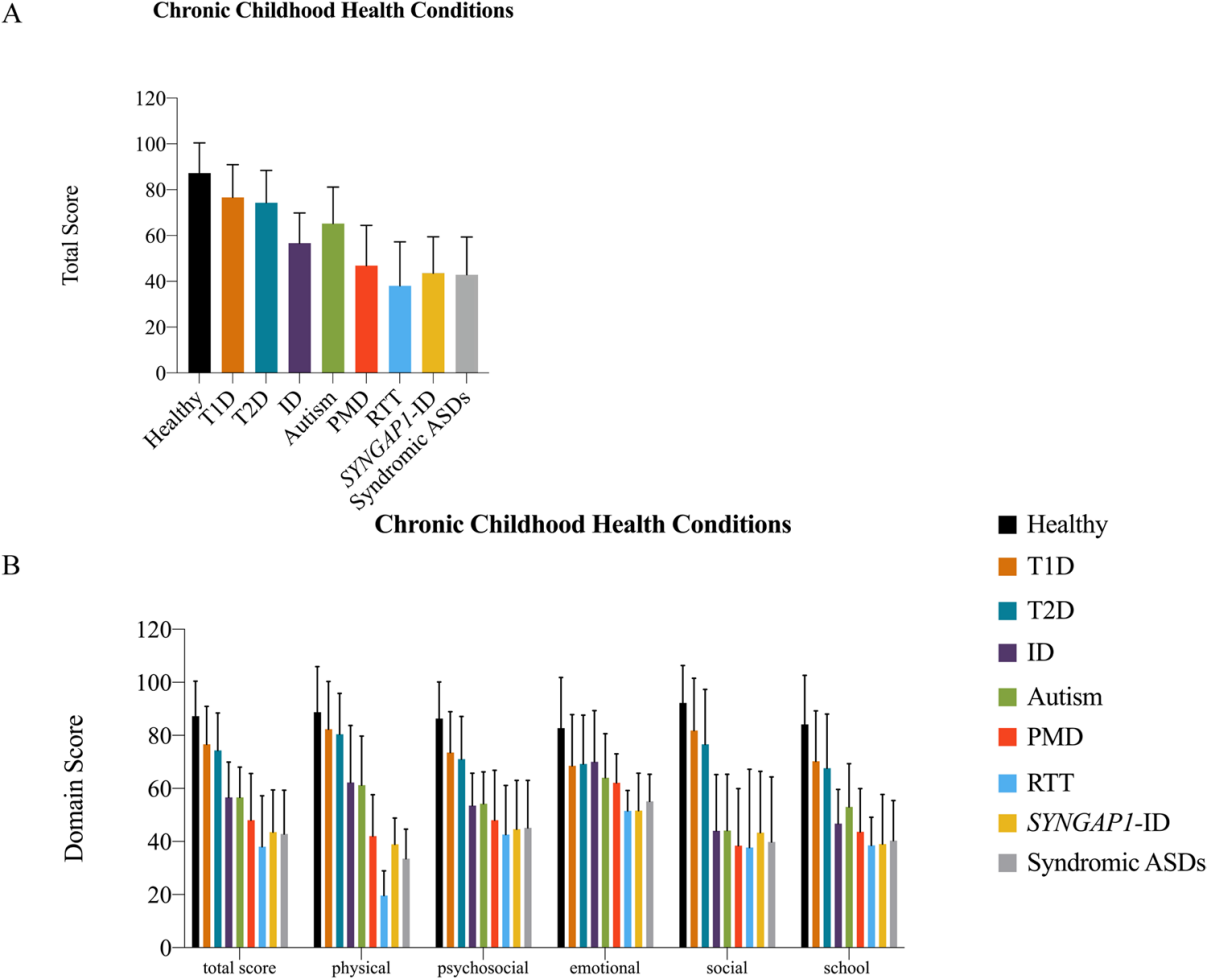
PedsQL of syndromic autism compared with other chronic childhood illnesses. **a** Total quality of life score and **b** domain scores for PedsQL across chronic childhood disorders

Chronic illnesses in children significantly impact HRQoL for both patients as well as caregivers. We hypothesized that the often complex phenotypes associated with these genetic syndromes would lead to worse HRQoL than other chronic childhood conditions. Therefore, we compiled PedsQL scores from published datasets in which similar age of inclusion criteria was implemented. Raw PedsQL score data from healthy controls, type 1 (T1D) and type 2 diabetes (T2D) (Varni et al., 2019), intellectual disability (ID) (Kilincaslan et al., 2019), and idiopathic autism (Stokes et al., 2017) were utilized as comparative assessment metric. For ease of interpretability, our PMD, RTT, and *SYNGAP1-*ID data were aggregated to create a new syndromic ASD cohort. A comparison of these total PedsQL cumulative scores across published datasets (Fig. 3a) revealed highest scores in HRQoL among healthy individuals (M = 87.2), followed by type I diabetes (M = 76.61), type II diabetes (M = 74.36), autism (M = 65.2), ID (M = 56.62), and lowest in our genetic disorders (M = 42.80) (p = 0.0004). In comparing each dimension measured by the PedsQL among the chronic childhood disorders, syndromic autism consistently scored lower than other chronic childhood illnesses (Fig. 3b).

## Discussion

Through a validated and simple to administer survey, we discovered that children with genetic disorders which strongly predispose to syndromic autism have significantly lower health-related quality of life compared with their neurotypical peers or children with other chronic health disorders. Among the syndromic autisms surveyed, we found that Rett syndrome had the lowest overall health-related quality of life based upon the total score from the PedsQL survey driven primarily by lower physical functioning. Surprisingly, each genetic disorder had a lower HRQoL score than idiopathic, non-syndromic autism.

When comparing individual domains of RTT, PMD and *SYNGAP1*-ID with previously published data for idiopathic autism, we found similar scores in emotional, social and school performance. Interestingly, the most significant difference between our genetic disorders and idiopathic autism lies in the physical domain measured by the PedsQL. While this is driven by a significant degree by Rett syndrome, we do see greater reported impairment for Phelan-McDermid syndrome and *SYNGAP1*-ID in physical functioning compared with idiopathic autism as well.

We also found that ASDs not only affect the individual, but the entire family. Children with an ASD tend to report higher anxiety levels than neurotypical children and exhibit greater prevalence of co-occurring internalizing (depressed mood and anxiety) and externalizing (hyperactivity and aggression) behavior problems (Rodriguez et al., 2019). There is an elevated risk for parents (caregivers) of children with an ASD to experience mental health problems, such as stress, anxiety, and depression, compared to parents of children without an ASD or the general population. From clinical and transactional models, we know that there is a continuous reciprocal interaction process between children and caregivers. ASD symptoms, including behavioural dysregulation, contribute to increased stress in the parent (caregiver), which may impact the parents’ QOL and inadvertently alter parenting behaviours in ways that reinforce the child’s behaviour problems and ASD symptoms, which can further influence the child’s QOL (Rodriguez et al., 2019). By viewing this from a transactional family dynamics perspective, we can see that ASDs not only affect the child but the whole family. Further studies addressing areas of improvement for families of children with syndromic ASDs regarding access to medical care, along with the support received by individuals with ASD may prove effective in addressing FQOL improvement.

Annual medical expenditures for children with ASD are four to six times greater than neurotypical children. It has been reported that the additional mean costs of caring for children diagnosed with ASD, including health care, education, ASD-related therapy, family-coordinated services and caregiver time total $17,081 per year (Lavelle et al., 2014). Applying these estimates to the projected 1.7% of children aged 3 to 17 years with ASD in the U.S. results in a total societal cost of $268 billion as of 2015. The societal cost is forecasted to increase to $461 billion by the year 2025 (Kogan et al., 2018). Similar economic modeling has not been performed for syndromic autisms. Given the greater concerns for health-related quality of life in syndromic autism compared with idiopathic autism, the costs per family associated with caring for children with syndromic autism spectrum disorders is presumptively even greater.

This work will aid in identifying therapeutic endpoints for evaluating treatment and intervention efficacy. This is true of both targeted therapy modalities such as physical, language and occupational therapies as well as therapies specifically targeting core autism symptoms such as applied behaviour analysis (ABA). From our data, physical impairments are a significant source of reduced quality of life for children with syndromic autism that is potentially overlooked in routine care. This research provides critical evidence, which might inform policymaking decisions regarding reconsideration of existing interventions aimed at patients with syndromic autism.

In addition to aiding clinicians in referring children with our genetic disorders to established therapeutic interventions, this work provides a framework for future clinical trials. Many clinical trials for neurodevelopmental disorders have failed in recent years (Jeste & Geschwind, 2016). One hypothesis regarding these failures is that previous trials have been hampered with clinical endpoints (i.e., measurements of cognition) that are difficult to attain improvement in the limited timeframe of typical clinical trials. Health-related quality of life instruments provide a more holistic measurement of child health for clinical trials, that are arguably more robust than any instrument that measures a single neurodevelopmental variable.

This work has limitations that require future investigation. First, because of the anonymous nature of this study, the genetic diagnoses of participants could not be verified. Second, we focused on three genetic diagnoses that strongly predispose to syndromic autism. Whether this is representative of all syndromic autisms is unclear and warrants further investigation. Third, age and socio-demographic variables were not collected in this online survey and as such could not be evaluated as variables in the HRQoL scores. This means that results of this study are difficult to generalize as well as it might be challenging to compare reported results across studies. Finally, as patients were largely recruited from patient-advocacy foundations, medical records were not available for review. Thus, we cannot say with certainty which individuals are clinically diagnosed with autism. Future studies investigating correlation of HRQoL with additional co-variables such as age, congenital malformations, epilepsy and degree of intellectual disability will be informative.

## APPENDIX A

### Phelan-McDermid Syndrome

Phelan-McDermid syndrome (PMD) is due to either a chromosome 22q13 microdeletion or *SHANK3* loss-of-function mutations. PMD is characterized by developmental delay, impaired speech, dysmorphic features, intellectual disability, epilepsy and occasionally congenital cardiac or renal abnormalities. It is one of the most common monogenic forms of ASD (Reierson et al., 2017) with approximately 84% of children with PMD fulfilling criteria for ASD (Soorya et al., 2013). Although the exact prevalence of PMD is unknown, at least 1200 cases have been reported world-wide according to the Phelan-McDermid Syndrome Foundation, and PMD is considered to be a relatively common cause of intellectual disability, accounting for between 0.5% and 2.0% of cases.

Onset of symptoms occur during the second year of life with delays in acquisition of language and social skills. While developmental delays may not be apparent in the first 12 months of life, hypotonia is often present in infants, contributing to poor feeding, weak cry, and poor head control. Regression, the loss of acquired skills, is a recognizable feature of PMD (Baird et al., 2008) with regression of language being most common. Epilepsy is diagnosed in over 30% of children with PMD with a wide spectrum of severity (Holder & Quach, 2016). No quantitative evaluation of the impact of this syndromic autism on health-related or family quality of life has ever been undertaken.

### Rett Syndrome

Rett syndrome (RTT) is an X-linked dominantly inherited, progressive neurodevelopmental disorder, characterized by apparently normal psychomotor development during the first 6 to 18 months of life, followed by regression that affects speech, motor skills, and purposeful hand function (Hagberg et al., 2002). It is the second leading cause of intellectual disability in girls, with an incidence ranging from 1/10,000 to 1/15,000 females born (Vignoli et al., 2010). About 80% of females with classic RTT and 25 to 75% of those with variant forms, depending on the criteria used for diagnosis and size and age of sampled population, have mutations in *MeCP2* (methyl CpG binding protein 2), which encodes a chromatin binding protein (Amir et al., 1999). The most recently revised clinical diagnostic criteria include partial or complete loss of acquired purposeful hand skills, regression of spoken language, onset of gait abnormalities which can include impaired (dyspraxia) or absence of ability (apraxia), and stereotypies including hand wringing/squeezing, clapping/tapping, mouthing, and washing/rubbing automatisms (Neul et al., 2010). Additional characteristic features include postnatal deceleration of head growth, motor abnormalities, gait posture ataxia, breathing dysfunction, with seizures and breathing disturbances being the most detrimental clinical phenotype in RTT (Smeets et al., 2012).

Recent studies have evaluated the QOL in subjects with RTT and their caregivers using questionnaires such as the Italian version of the impact of childhood illness scale (Parisi et al., 2016). These studies have demonstrated a significant negative impact on both the child’s development and the entire family.

### SYNGAP1 Related Intellectual Disability

*SYNGAP1-*related intellectual disability (*SYNGAP1-*ID) is a neurological disorder characterized by moderate to severe intellectual disability evident in early childhood first described a decade ago. Early features consist of delayed speech and motor skills, with individuals typically having weak muscle tone (hypotonia), contributing greatly to difficulties with motor development (Mignot et al., 2016). Autism spectrum disorder is diagnosed in as many as 73% of individuals with pathogenic *SYNGAP1* mutations (Jimenez-Gomez et al., 2019). Moreover, in unbiased exome sequencing of over eleven thousand individuals with autism, *SYNGAP1* mutations were among the top three monogenic causes of autism (Ashitha & Ramachandra, 2020).

Early features of *SYNGAP1*-ID are usually present within the first two years of life, and typically include severely delayed development of language and motor skills. Children are often either non-verbal or minimally verbal. On average, they walk independently for the first time on average at 22 months of age, which is approximately double that of neurotypical children. Behavioural abnormalities may include hand flapping, obsessions with certain objects and poor social development. Other behavioural abnormalities include hyperactivity, impulsivity, physical aggression, mood swings, sullenness and rigidity. Greater than 90% of children with *SYNGAP1*-related intellectual disability have epilepsy which can be intractable and devastating (Jimenez-Gomez et al., 2019; Vlaskamp et al., 2019). To date, no studies investigating the impact on health-related or family quality of life have been conducted for *SYNGAP1*-related intellectual disability.

## APPENDIX B

See Table 8.

**Table 8:** Demographics of Patient Registries

	Phelan-McDermid syndrome (N = 452)	*SYNGAP1*-related intellectual disability (N = 150)
Child’s sex (female)	49.77%	56.98%
Child’s mean age	10.35	5.98
Private, military or other insurance	–	59.52%
White	51.34%	90%
Income < $50,000	–	50%
